# Neuronal population model of globular bushy cells covering unit-to-unit variability

**DOI:** 10.1371/journal.pcbi.1007563

**Published:** 2019-12-27

**Authors:** Go Ashida, Helen T. Heinermann, Jutta Kretzberg

**Affiliations:** Cluster of Excellence "Hearing4all", Department of Neuroscience, University of Oldenburg, Oldenburg, Germany; University of California at Berkeley, UNITED STATES

## Abstract

Computations of acoustic information along the central auditory pathways start in the cochlear nucleus. Bushy cells in the anteroventral cochlear nucleus, which innervate monaural and binaural stations in the superior olivary complex, process and transfer temporal cues relevant for sound localization. These cells are categorized into two groups: spherical and globular bushy cells (SBCs/GBCs). Spontaneous rates of GBCs innervated by multiple auditory nerve (AN) fibers are generally lower than those of SBCs that receive a small number of large AN synapses. In response to low-frequency tonal stimulation, both types of bushy cells show improved phase-locking and entrainment compared to AN fibers. When driven by high-frequency tones, GBCs show primary-like-with-notch or onset-L peristimulus time histograms and relatively irregular spiking. However, previous *in vivo* physiological studies of bushy cells also found considerable unit-to-unit variability in these response patterns. Here we present a population of models that can simulate the observed variation in GBCs. We used a simple coincidence detection model with an adaptive threshold and systematically varied its six parameters. Out of 567000 parameter combinations tested, 7520 primary-like-with-notch models and 4094 onset-L models were selected that satisfied a set of physiological criteria for a GBC unit. Analyses of the model parameters and output measures revealed that the parameters of the accepted model population are weakly correlated with each other to retain major GBC properties, and that the output spiking patterns of the model are affected by a combination of multiple parameters. Simulations of frequency-dependent temporal properties of the model GBCs showed a reasonable fit to empirical data, supporting the validity of our population modeling. The computational simplicity and efficiency of the model structure makes our approach suitable for future large-scale simulations of binaural information processing that may involve thousands of GBC units.

## Author summary

In the auditory system, specialized neuronal circuits process various types of acoustic information. A group of neurons, called globular bushy cells (GBCs), faithfully transfer timing information of acoustic signals to their downstream neurons responsible for the perception of sound location. Previous physiological studies found representative activity patterns of GBCs, but with substantial individual variations among them. In this study, we present a population of models, instead of creating one *best* model, to account for the observed variations of GBCs. We varied all six parameters of a simple auditory neuron model and selected the combinations of parameters that led to acceptable activity patterns of GBCs. In total, we tested more than half a million combinations and accepted ~11600 GBC models. Temporal spiking patterns of real GBCs depend on the sound frequency, and our model population was able to replicate this trend. The model used here is computationally efficient and can thus serve as a building block for future large-scale simulations of auditory information processing.

## Introduction

Processing of acoustic information along the auditory pathways is performed in a parallel and hierarchical manner. In the mammalian central nervous system, the cochlear nuclei are the first stations that receive sound information transferred from the auditory periphery [1]. Different types of neurons in the cochlear nuclei process and convey divergent features of the sound waveform [2]. Bushy cells in the anteroventral cochlear nucleus (AVCN) encode precise timing information of the sound stimulus, which is then sent to the superior olivary complex and used for binaural sound localization [3].

**Fig 1:**
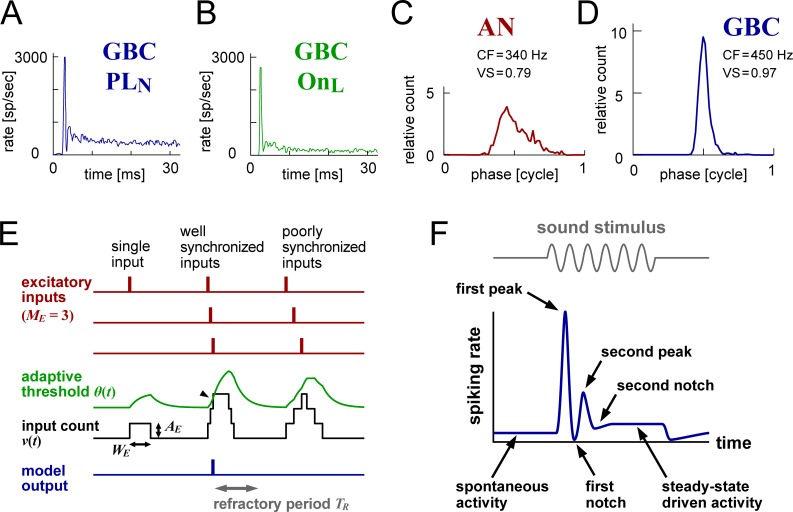
Empirical response patterns of GBC and schematic drawings of the model structure and PSTH output. **A-B:** PSTHs of cat GBCs: a PL_N_-type (**A**) and an On_L_-type (**B**). Adapted from [24]. **C-D**: Low-frequency phase histograms of a cat auditory nerve (**C**) and GBC (**D**). Adapted from [25]. CF: characteristic frequency; VS: vector strength. **E:** Operation of the adaptive coincidence counting model (see Materials and Methods for the equations). The model neuron receives *M*_*E*_ excitatory synaptic inputs (red) simulated by the AN model (*M*_*E*_ = 3 in this example). Each presynaptic input spike (small vertical red bar) induces a postsynaptic response of a length *W*_*E*_ and amplitude *A*_*E*_ in the model neuron (black rectangular bump on left). The adaptive threshold *θ*(*t*) (green), parameterized by a time scale *T*_*A*_ and strength *S*_*A*_, develops according to the summed input count *v*(*t*) (black). When the input count reaches or exceeds the threshold (arrowhead), an output spike is generated (blue). After each spike output, the model is in the refractory period of a duration *T*_*R*_ (gray), in which no further spikes can be generated. Insufficient synchrony of input spikes (three inputs on right) leads to a failure of output spike generation due to the adaptive threshold. **F:** Components of a primary-like-with-notch (PL_N_) peristimulus time histogram (PSTH) used for judging the plausibility of each instance of the modeled bushy cell. Depending on the parameters used, a second notch may or may not exist while the other components almost always appear.

Based on anatomical and physiological characteristics [1,4,5], bushy cells are subdivided into two groups: spherical bushy cells (SBCs) and globular bushy cells (GBCs). An SBC receives only few (typically 1–4 in cats [6,7]) large excitatory endbulb synapses from auditory nerves (ANs) [8,9], while a GBC has a larger number (~20 in cats [7,10]) of medium-sized synapses, called modified endbulbs [11,12,13]. SBCs send excitatory projections to the ipsilateral medial and lateral superior olive (MSO/LSO) as well as the contralateral MSO [14,15,16], which are the major brainstem structures responsible for binaural sound localization [3]. GBCs innervate several monaural and binaural nuclei in the superior olivary complex [16–19], including the ipsilateral lateral nucleus of the trapezoid body (LNTB) and the contralateral medial nucleus of the trapezoid body (MNTB), which provide inhibitory projections to MSO and LSO. When stimulated with high-frequency tones (>3 kHz), SBCs show "primary-like (PL)" peristimulus time histograms (PSTHs) that resemble those of AN fibers [15,20], and GBCs usually present "primary-like-with-notch (PL_N_)" PSTHs having a short pause (~1 ms) of spiking after pronounced onset activity (Fig 1A) [12,19,21–24]. A smaller number of GBCs show "onset-L (On_L_)" PSTHs, which resemble PL_N_ PSTHs but with lower sustained activity (Fig 1B) [12,19,22–24]. For low-frequency stimulation (<1 kHz), both types of bushy cells show enhanced phase-locking and entrainment compared to ANs (Fig 1C and 1D) [25–28]. Enhancement of temporal properties, however, might be caused through different biophysical mechanisms between SBCs and GBCs [29].

In this study, we focus on GBCs, in which the convergence of multiple AN inputs substantially contributes to the improvement of phase-locking and entrainment [30–34]. In order to simulate and investigate the acoustic information processing in GBCs, we aim to construct a computational model that can replicate known physiological response characteristics of GBCs. Previous electrophysiological data obtained *in vivo*, however, presented considerable variations among units (e.g., [25,35–38]), making it difficult to create a single "gold standard" model that would fit all available data. Thus our goal here is to construct a population of models that can account for the observed unit-to-unit variations in spontaneous and sound-driven activity of GBCs. A similar approach of generating a database of models, which is sometimes called "neuronal population modeling", has been shown to be useful in studying the functional roles of various ion channels and their interplay in non-auditory systems [39,40,41] (see [42] for a review).

Physiological single neuron models can be classified into several categories (see [43,44] for reviews of representative classes). Prior models of bushy cells include shot-noise models that have a simplified description of synaptic inputs and focus primarily on the input-output relations [25,45–48], integrate-and-fire-type models that have a membrane potential and an abstracted spike-generation mechanism [33,47,49,50], and more complex Hodgkin-Huxley-type models that deal with detailed biophysical functions of ionic conductances [30,31,46,51–54]. Simple models are generally suitable for computationally efficient simulations and mathematical analyses, while complex models are often required for the investigation of detailed mechanisms underlying spiking phenomena [44,55]. These different model types can complement each other to advance our understanding in computational biology [55]. In this study, we use a modified version of the coincidence counting model (Fig 1E), a member of shot-noise models [55], whose computational simplicity allows us to simulate a large number of model neurons within a reasonable computational time. Similar models have been used for examining the temporal coding in binaural brainstem neurons that receive inputs from the cochlear nucleus [56,57].

In the following sections, we first present representative response patterns of the modeled GBC, using a fixed combination of model parameters. Next, we systematically vary the parameters to create a population of models, examine the behavior of each model using a limited variety of tonal stimuli, and select models that produce physiologically plausible responses. We then analyze the accepted parameter combinations and validate the resulting model GBC population under a wider range of stimulus conditions. Limitations and applicability of our model framework are addressed in the Discussion section. We expect that the population of computationally efficient GBC models developed in this study will serve as a building block for future large-scale simulations of auditory information processing.

## Results

### Overview: Model structure and target properties

#### Adaptive coincidence counting model

To simulate the spiking activity of GBCs, we used a modified version of the coincidence counting model [56,57] extended with an adaptive threshold (Fig 1E). Each input from AN fibers (red) is converted into a rectangular postsynaptic input count (black). The threshold of the model (green) varies according to the preceding input level. The model neuron generates an output spike (blue) when the number of input counts reaches or exceeds the threshold (small arrowhead). After generating an output spike, the model is in an absolute refractory period, in which no more spikes are produced. We refer to this model as the "adaptive coincidence counting model". AN inputs to the GBC model were simulated with the established auditory periphery model by Zilany, Bruce and others [58–61].

The adaptive coincidence counting model has six parameters (Table 1): the number of AN inputs *M*_*E*_, duration *W*_*E*_ and amplitude *A*_*E*_ of the excitatory input, refractory period *T*_*R*_, and the time scale *T*_*A*_ and strength *S*_*A*_ of threshold adaptation. The duration of the excitatory input, *W*_*E*_, is also called the "coincidence window" [57], because coincident arrivals of incoming presynaptic spikes within this time frame are required to generate an output. In the following text, we refer to an individual realization of the model with each specific combination of parameters as a "model instance" (or simply an "instance"), to distinguish it from the general model framework and from real neuronal units. In this study, we varied all six parameters in the range shown in Table 1 to generate a population of models (567,000 instances in total) and selected instances that satisfied our physiological criteria for GBCs (explained below).

**Table 1:** Parameter ranges. The total number of parameter combinations is 7×10×9×9×10×10 = 567,000. The excitatory input amplitude does not have a unit, as it is defined as the relative amplitude with respect to the static threshold of *θ*_*S*_ = 1. See "Adaptive coincidence counting model" and "Selection of model instances" in Materials and Methods for detailed explanations of the parameters and their ranges used. The parameter values used for Figs 2, 3, 4, 12A–12C, 13A–13C and 14 are shown in bold.

Parameter	Values
Number of excitatory inputs *M*_*E*_	9, 12, 16, **20**, 25, 30, 36
Coincidence window [ms] *W*_*E*_	0.08, 0.16, 0.24, **0.32**, 0.40, 0.48, 0.56, 0.64, 0.72, 0.80
Excitatory input amplitude *A*_*E*_	0.24, 0.28, 0.32, 0.36, **0.40**, 0.44, 0.48, 0.52, 0.56
Refractory period [ms] *T*_*R*_	0.70, 0.80, 0.90, 1.00, 1.10, **1.20**, 1.30, 1.40, 1.50
Adaptation time constant [ms] *T*_*A*_	0.05, 0.10, 0.15, 0.20, **0.25**, 0.30, 0.35, 0.40, 0.45, 0.50
Adaptation strength *S*_*A*_	0.40, 0.50, 0.60, 0.70, **0.80**, 0.90, 1.00, 1.10, 1.20, 1.30

#### Selection of model instances

Physiological responses of GBCs are characterized by low spontaneous rates [15,22,23,38,62], PL_N_-type PSTHs [12,19,22–24] and irregular firing [30,36] for high-frequency tone bursts, and enhanced phase-locking and entrainment for low-frequency tonal stimulation [25–28]. Based on these response characteristics, we defined a set of target properties that a model GBC instance had to satisfy. Without sound stimulus, (1) the spontaneous spiking rate should be lower than 30 (spikes/sec). For high-frequency stimulation (7000 Hz, 70 dB SPL), (2a) the sound-driven steady-state spiking rate should be higher than 150 (spikes/sec), (2b) the coefficient of variation of interspike intervals (ISIs) should be between 0.65 and 0.95, and (2c) the PSTH should have a PL_N_ shape (Fig 1F). For low-frequency stimulation (350 Hz, 70 dB SPL), (3a) the vector strength (VS) should be higher than 0.9 and (3b) the entrainment index (EI) should be higher than 0.9. Both VS and EI are measures of temporal spiking patterns but with distinct definitions: a high EI requires nearly one-to-one correspondence between the spiking rate and stimulus sound frequency, whereas a high VS can be achieved even with lower spiking rate as long as the spike timings are well aligned with a certain stimulus phase. See Materials and Methods for the complete definition and relevant references of each output measure; limitations of these criteria will be addressed in the Discussion section.

Whereas a majority of GBCs have PL_N_-type PSTHs, a smaller fraction of GBCs show "Onset-L (On_L_)"-type PSTHs, in which a sharp onset peak is followed by a low sustained spiking rate [12,19,22–24]. Sometimes PL_N_ units and On_L_ units are not clearly distinguished and are instead collectively grouped as PL_N_ (e.g., [38,63]; see discussion in [36]). In the present study, the model instances that had (2a') a lower steady-state spiking rate (50–150 spikes/sec) and satisfied all other response criteria above (1, 2b, 2c, 3a, 3b) were classified as On_L_. We analyzed PL_N_ instances and On_L_ instances separately.

#### Validation of model instances

After selecting model instances using the aforementioned criteria, we validated the model population with a wider variety of sound stimuli. We varied the tonal stimulus frequency between 200 and 5000 Hz, calculated the vector strength and entrainment index of each selected instance at each of these frequencies, and compared the population response with empirical data [25]. We also drove the model GBC instances with high-intensity, low-frequency tones to obtain so-called "tail-sync" responses, in which GBCs that are tuned to medium or high frequencies (> 2kHz) show prominent phase-locking at frequencies typically below 1 kHz [63]. We then stimulate the GBC instances with sinusoidally amplitude-modulated (SAM) tones to examine the phase-locking of high-frequency units to low-frequency envelopes [64–66]. In the following sections, we first show the responses of a representative GBC model instance using one fixed set of model parameters (Table 1, numbers in bold). Next, we vary the parameters to study the relationship between the model parameters and output measures. Then we validate the outcome of the selected model population using empirical data of GBC responses.

### Representative model responses

#### General activity patterns of a PL_N_ instance

**Fig 2:**
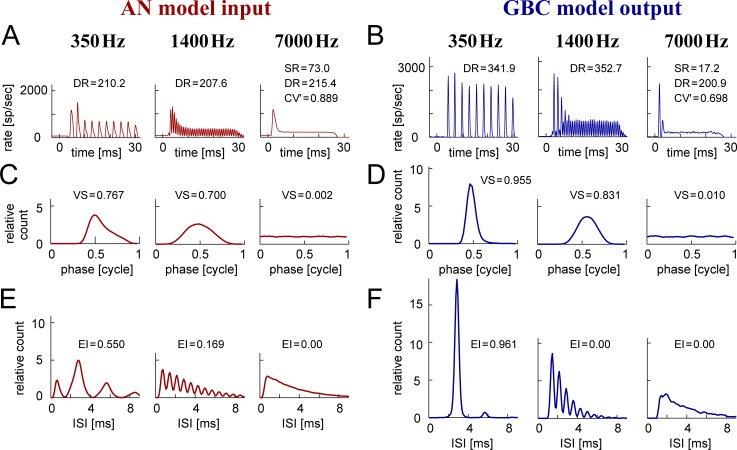
Response patterns of the model input and output. **A:** PSTHs of the AN model. **B:** PSTHs of a representative GBC model instance that receive the AN model inputs. **C:** Period histograms of the AN model. **D:** Period histograms of the GBC model. **E:** ISIHs of the AN model. **F:** ISIHs of the GBC model. Each column corresponds to either low- (350 Hz), medium- (1400 Hz), or high-frequency (7000 Hz) tonal stimulation. The sound intensity was fixed at 70 dB SPL. SR: average spontaneous rate (in spikes/sec); DR: average sound-driven rate (in spikes/sec); CV': modified coefficient of variation; VS: vector strength; EI: entrainment index; see Materials and Methods for their definitions.

Fig 2 shows representative response patterns of the AN model and the GBC model driven by low- (350 Hz), medium- (1400 Hz) and high-frequency (7000 Hz) tones. The number of AN inputs to this GBC model instance was fixed to 20, and the other parameter values (Table 1, bold numbers) were chosen as the median values of the admitted PL_N_ population with *M*_E_ = 20 inputs. At all frequencies tested, the PSTHs of the GBC model showed sharper peaks (Fig 2B) than those of the AN model (Fig 2A). At the high frequency (7000 Hz), the GBC output presented a notch after the onset peak in the PSTH, while the AN input showed a smooth, gradual decrease after the onset. The steady-state responses of the AN and GBC models to high-frequency sounds were relatively irregular, with a modified coefficient of variation (CV') of ISIs over 0.65, matching previous *in vivo* measurements [30]. The average spontaneous rate of the GBC model (below 30 spikes/sec) was much lower than the AN spontaneous rate (~70 spikes/sec), because synchronized arrivals of multiple inputs are required to generate a spike in the GBC model.

In accordance with the sharpened PSTHs, phase (Fig 2C and 2D) and interspike interval histograms (ISIHs; Fig 2E and 2F) also showed sharper peaks for the GBC output than the AN input. In response to the low-frequency stimulation (350 Hz), the GBC output presented a marked enhancement of phase-locking (Fig 2D, left) and entrainment (Fig 2F, left). This enhancement is explained by the convergence of multiple AN inputs [30–34]. For the low-frequency stimulus, the ISIH of the GBC model showed a pronounced peak at around ~2.9 ms (reciprocal of 350 Hz) (Fig 2F, left), while the AN model had multiple sidepeaks (Fig 2E, left). The lower sidepeak below 1 ms was due to the occurrence of multiple spikes in one tonal cycle, which was eliminated in the GBC model because of the refractory period and threshold adaptation. The second and third sidepeaks around 5.7 ms and at 8.6 ms were due to skipping cycles, which were also reduced in the GBC model. The synchrony of the converging AN input fibers was sufficient to evoke a GBC spike in every tonal cycle; consequently, the driven activity of the GBC model (341.9 spikes/sec) was highly entrained to the stimulus frequency of 350 Hz. Entrainment of the GBC output diminished for frequencies at 1400 kHz (Fig 2F, center), since the ISI of the model GBC could not be smaller than the refractory period (1.2 ms in this example).

#### Level-dependent activity

**Fig 3:**
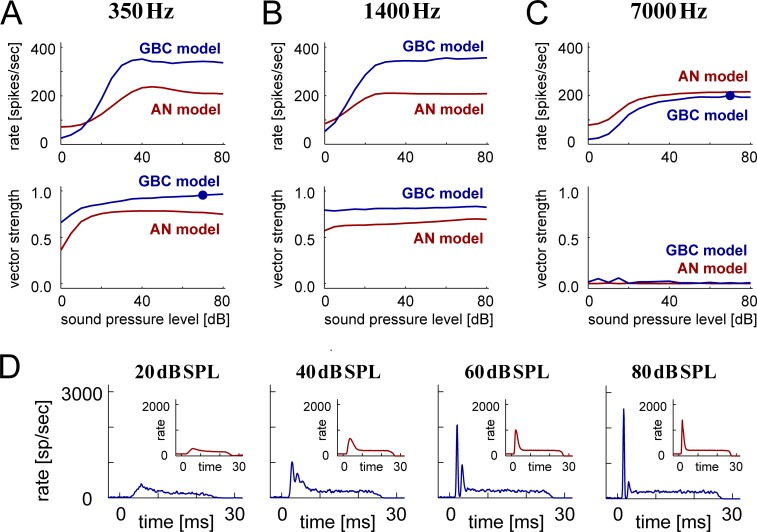
Level-dependent activity of model input and output. **A-C:** Level-dependent spiking rates (top) and phase-locking (bottom) of AN (red) and GBC (blue) models driven by low (**A:** 350 Hz), medium (**B:** 1400 Hz), and high (**C:** 7000 Hz) frequency tonal stimulation. Filled circles in **A** (bottom) and **C** (top) indicate the responses at 70 dB SPL, the level at which the GBC model was further tested. **D:** Level-dependent PSTH shapes of the AN (red inset) and GBC (blue) models for 7000 Hz tonal stimulation.

Fig 3 presents the level-dependence of the spiking activity of the AN and the GBC model. Simulated spiking rates almost monotonically increased with level and saturated over 40 dB SPL (Fig 3A–3C, top), while the degrees of phase-locking measured as VS were stable above 20 dB SPL. The PSTH shape of the AN model were relatively unaffected with the level (Fig 3D, red inset). In contrast, the GBC model (Fig 3D, blue) gradually changed its PSTH shape from PL to PL_N_ with an increasing sound level. A similar transition was observed in previous *in vivo* recordings [19,24,36]. Based on these observations, we fix the sound level at 70 dB for tonal stimuli to obtain stable results in the following simulations (see Discussion for possible limitations of using one fixed sound level).

#### Frequency-dependent activity

**Fig 4:**
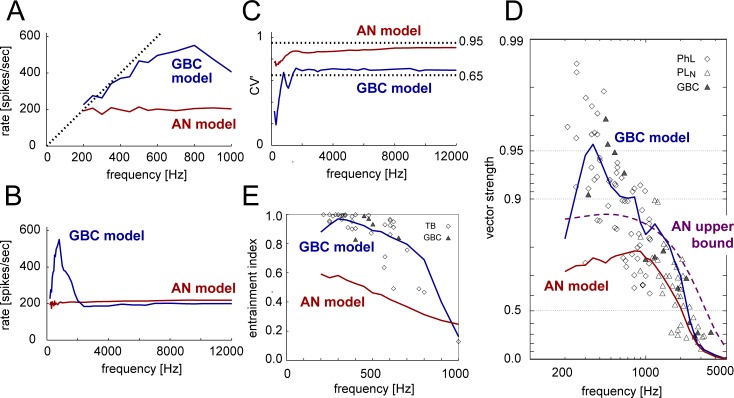
Frequency-dependent activity of model input and output. **A:** Spiking rates for tonal stimulation below 1 kHz. Dotted line shows the spiking rate being equal to the stimulus frequency. **B:** Spiking rates for tonal stimulation between 0 and 12 kHz. **C:** Coefficients of variation of the interspike intervals. Vertical dotted lines show empirical lower and upper bounds for bushy cells [30]. **D:** Vector strength (VS) plotted along the scale of log(1-VS). Symbols show experimentally observed phase-lockers (PhL, diamond: units with multiple peaks in their PSTH that lock to the stimulus frequency; they are most likely to be SBCs or GBCs but possibly other cell types), PL_N_ units (unfilled triangle: presumed to be GBCs), and histologically confirmed GBCs (filled triangle). **E:** Entrainment index. Symbols show fibers recorded in the trapezoid body (TB, diamonds: most likely to be SBCs or GBCs but possibly other cell types) and histologically confirmed GBCs (filled triangle). Red and blue lines in **A-E** show AN and GBC model responses, respectively. The empirical data reported in [25] were used for the AN upper bound of VS (dashed purple curve in **D**) and data points in **D-E**.

The sound-driven activity of the GBC model was entrained to low-frequency tones up to about 600 Hz (Fig 4A, blue), while the spike rates of the AN model were mostly unchanged with frequency (Fig 4A, red). This trend corresponds to the frequency-dependent change of the entrainment index (Fig 4E). The output rates of the GBC model at mid-to-high frequencies (>2000 Hz) were almost constant (Fig 4B). The CV' values of the GBC for frequencies over 2000 Hz was consistently higher than 0.65 (Fig 4C), indicating relatively irregular spiking outputs [30]. Phase-locking and entrainment of the GBC model were most prominent for 300–400 Hz (Fig 4D and 4E) and gradually decreased with frequency. The GBC model output presented better phase-locking than its AN input up to about 3000 Hz (Fig 4D, blue and red). For frequencies below 1000 Hz, phase-locking of the GBC model (Fig 4D, blue) was even better than the measured upper bound of AN fibers (Fig 4D, purple). Such enhancement of low-frequency phase-locking was reported in various animals (cats [25,27]; rats [26]; chinchillas [27]; gerbils [28]). At very low frequencies (200–300 Hz), VS of the GBC model was lower than the peak value at 350 Hz, because of multiple spike generation in one tonal cycle (see [28] for a review and discussion on such peak-splitting).

### GBC population—Model selection

#### Selecting PL_N_ model instances

**Fig 5:**
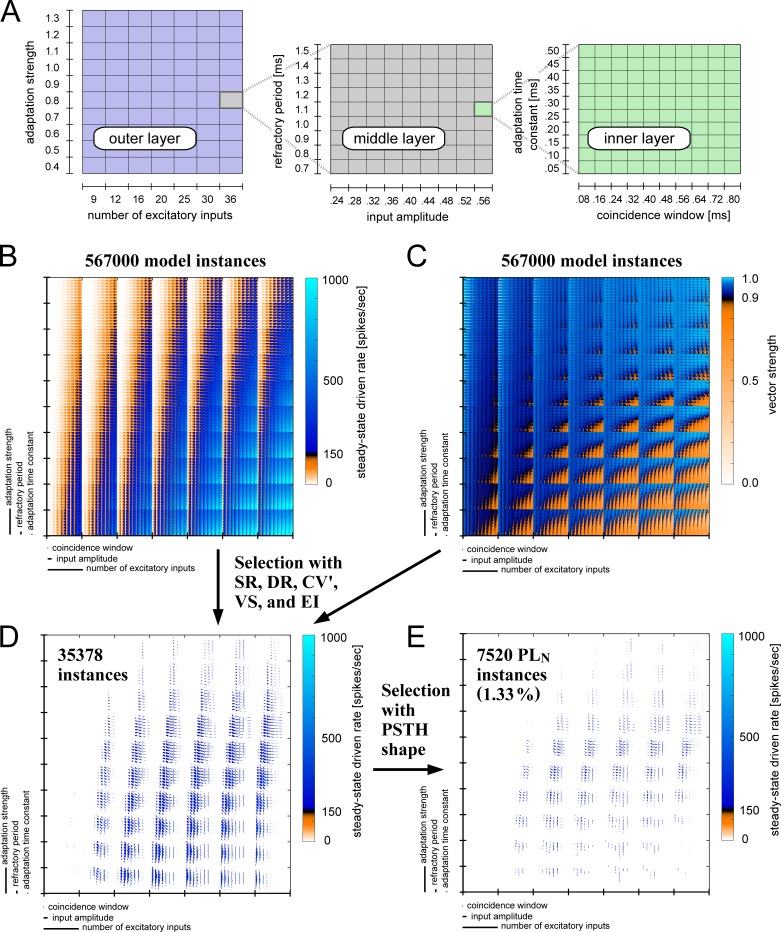
Dimensional stacking plots of the model parameter space. **A:** Schematic presentation of dimensional stacking. Six-dimensional parameters are nested into three layers of two-dimensional spaces. Each rectangular cell of the outer layer, which corresponds to a specific combination of two parameters (number of inputs *M*_*E*_ and adaptation strength *S*_*A*_, in this case), represents a middle layer (input amplitude *A*_*E*_ and refractory period *T*_*R*_), which, in turn, consisted of the cells of the inner layer (coincidence window *W*_*E*_ and adaptation time constant *T*_*A*_). **B:** Steady-state driven rates (7000 Hz pure tone, 70 dB SPL) of all 567,000 model instances shown with dimensional stacking. The spiking rate threshold of 150 (spikes/sec) is shown in black: instances that had lower spiking rates than this threshold (white-orange) were discarded, while instances with spiking rates equal to or higher than this threshold (dark to light blue) were adopted for further analyses. **C:** Degree of phase locking quantified as VS (350 Hz pure tone, 70 dB SPL) of all model instances. The VS threshold of 0.9 is shown in black: instances that showed lower VS than this threshold (white-orange) were discarded and those with higher (blue) were accepted. **D:** Steady-state driven rates of model instances that satisfied all the criteria of spontaneous activity, high-frequency sound-driven activity (rate and coefficient of variation) and low-frequency sound-driven activity (phase-locking and entrainment). **E:** Steady-state driven rates of model instances that satisfied all the criteria in **D** and additionally showed PL_N_-type PSTHs. In **D** and **E**, discarded model instances are shown in white, while accepted models are shown by the same color code as in **B**.

Whereas our GBC model with the default parameters was able to reproduce typical bushy cell response characteristics, reported GBC activity *in vivo* (e.g., data points in Fig 4D) show considerable variations among units. To account for this unit-to-unit variability, we varied all six model parameters and created over half a million model instances (Table 1). As described briefly in a previous section titled "Selection of model instances" and more thoroughly in Materials and Methods, we systematically tested each instance of this population with its spontaneous and sound-driven activity. Here we use a method called "dimensional stacking" [67,68] to visualize the 6-dimensional parameter space in 2-dimensional plots. In a dimensional stack, two parameter dimensions are selected for each layer and the layers are nested in one another (Fig 5A). The order of the dimensions was manually selected so that the trends of parameter-dependent responses could be seen in the resulting images.

**Fig 6:**
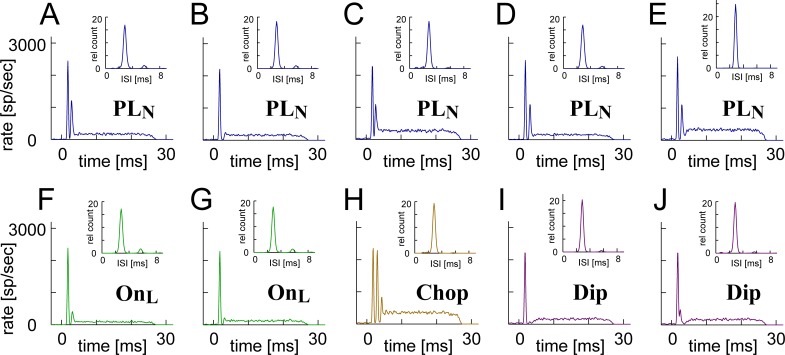
Representative high-frequency tone-driven PSTHs (7000 Hz, 70 dB SPL) and low-frequency tone-driven ISIHs (350 Hz, 70 dB SPL, insets) of model instances. **A-E:** PL_N_ instances that were included in our GBC model population. The second peak may be large (**A**) or small (**B**). Steady-state rates may also be high (**C**) or low (**D**), with other properties being almost identical. Some PL_N_ instances show a very large peak in ISIH, indicating pronounced phase-locking and entrainment (**E**). **F-J:** Non-PL_N_ instances including: On_L_ units having pronounced onset activity followed by a low sustained rate (50–150 spikes/sec, **F-G**), chopping response with multiple onset peaks and notches (**H**), and a dip in PSTH having a long (> 2 ms) pause after the first (**I**) or second onset peak (**J**). Parameter values used for each panel are shown in Table 2.

High-frequency sound-driven spiking rates of the population are presented in Fig 5B. The light blue areas near the bottom-right corner indicate that the spike rate became high for a large number of synaptic inputs combined with weak adaptation. Vertically arranged stripes of the middle layer (in each of the 7 × 10 rectangles) show that the driven rate was affected also by the input amplitude but not by the refractory period. The degrees of phase-locking (VS) to low-frequency sound is shown in Fig 5C. The brightest blue areas in the top-right corner suggest that a combination of strong adaptation and many converging inputs is required for a prominent enhancement of phase-locking with VS > 0.95. In contrast, insufficient adaptation led to excessive spiking activity and thus to degraded phase-locking (Fig 5C, orange areas). In a later section, we present further quantitative analyses of the dependence of the output measures on the model parameters. Applying the thresholds for spontaneous and sound-driven activity, we selected 35378 candidate instances (Fig 5D). We then analyzed their PSTH shapes and obtained a population of 7520 model instances that satisfied all of our criteria (see "PSTH shapes" in Materials and Methods) to be accepted as a GBC-like, PL_N_-type unit (Fig 5E). The resulting PL_N_ model instances still showed some variation in the shapes of PSTH and ISIH (Fig 6A–6E).

Model instances that had low sustained spiking rate (50–150 spikes/sec) and satisfied all the other PL_N_ criteria were categorized as On_L_ units (Fig 6F and 6G), which we adopted for further analyses (4094 model On_L_ instances in total). The candidate instances whose PSTHs did not meet our PL_N_/On_L_ criteria included choppers (Fig 6H) and dipper units (Fig 6I and 6J). Chopping responses with multiple peaks and troughs at the onset whose intervals were irrelevant to the stimulus frequency were typical for stellate cells but not for bushy cells in the cochlear nucleus [2,22]. In our simulations, instances that had no or weak threshold adaptation tended to be choppers. Dip-type responses with a relatively long (2–10 ms) pause after the onset were also atypical for bushy cells [30]. Units with a long integration time (i.e., long coincidence window) and/or long adaptation time constant often showed a dip in their PSTHs, because of the long-lasting threshold augmentation after onset. Choppers and dippers were excluded from further analyses below, even though some of them presented an enhancement of VS and EI (Fig 6H–6J, inset) compatible to that of GBCs (see Discussion). In the population of candidate GBC instances (Fig 5D), we found no PSTHs that were clearly classified as PL (without a notch) at 70 dB SPL. The absence of PL responses corresponds to previous modeling results that convergence of multiple inputs generally leads to a notch after onset [30].

**Table 2:** Parameter values used for Fig 6.

Figure	6A	6B	6C	6D	6E	6F	6G	6H	6I	6J
Number of excitatory inputs *M*_*E*_	20	20	20	20	36	20	20	20	20	20
Coincidence window [ms] *W*_*E*_	0.24	0.32	0.48	0.56	0.40	0.40	0.32	0.56	0.40	0.40
Excitatory input amplitude *A*_*E*_	0.32	0.44	0.48	0.28	0.32	0.32	0.40	0.32	0.48	0.48
Refractory period [ms] *T*_*R*_	1.00	1.40	0.90	1.30	1.20	1.20	1.20	1.20	1.20	0.70
Adaptation time constant [ms] *T*_*A*_	0.50	0.20	0.15	0.30	0.30	0.25	0.25	0.25	0.30	0.30
Adaptation strength *S*_*A*_	0.50	1.00	0.90	0.60	0.80	0.80	1.00	0.40	1.20	1.20

#### Parameter distribution in the 6-dimensional space

**Fig 7:**
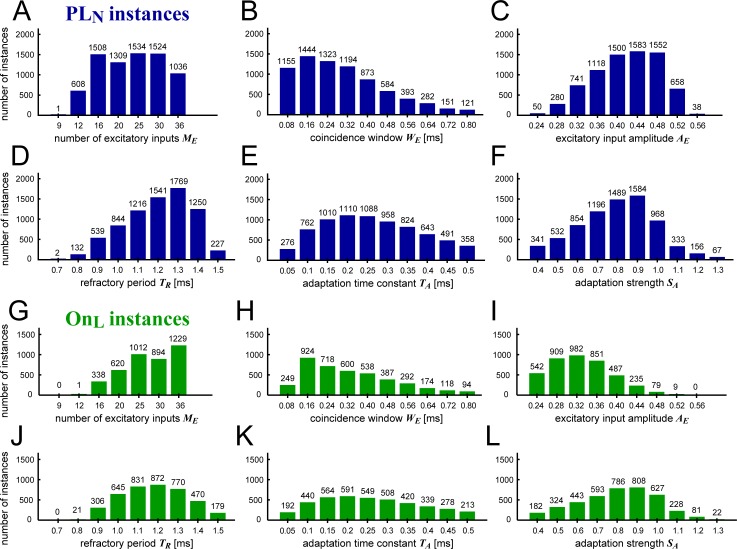
Distribution of parameters for accepted GBC models. **A-F:** PL_N_-type instances. **G-L:** On_L_-type instances. **A,G:** Number of excitatory inputs *M*_*E*_. **B,H:** Coincidence window *W*_*E*_. **C,I:** Excitatory input amplitude *A*_*E*_. **D,J:** Refractory period *T*_*R*_. **E,K:** Time constant of adaptation *T*_*A*_. **F,L:** Strength of adaptation *S*_*A*_. The total number of model instances in each plot is 7520 (PL_N_) or 4094 (On_L_).

Fig 7A–7F shows the distribution of each model parameter value for the accepted 7520 PL_N_ instances. The number of excitatory inputs distributed relatively uniformly (Fig 7A, 12–36 inputs), compared to other parameters having unimodal shapes (Fig 7B–7F). For *M*_*E*_ = 9 inputs, only one instance was accepted as a PL_N_ unit. We note that, when the criterion for the spontaneous rate was loosened (e.g., from 30 to 50 spikes/sec), more instances with small numbers of inputs were accepted. The distributions of the other five parameters were skewed, showing a longer tail in one direction than in the other (Fig 7B–7F). The length of the coincidence window *W*_*E*_ (= duration of excitatory inputs) had a peak count around 0.16 ms and a long tail up to 0.80 ms (Fig 7B). The adaptation time constant *T*_*A*_ (Fig 7E) had a broad distribution, indicating that this parameter may not have a strong effect on the resulting model outcome. In contrast, the excitatory input amplitude *A*_*E*_ (Fig 7C), refractory period *T*_*R*_ (Fig 7D), and adaptation strength *S*_*A*_ (Fig 7F) showed a mild limit, above which the number of accepted instances dropped substantially. This implies that these limits can be related to the limits of our criteria used for selecting PL_N_ units. A long refractory period (*T*_*R*_ = 1.5 ms), for example, often led to more regular spiking (CV' < 0.65) than empirical GBC data [30]. Instances with a large input amplitude (*A*_*E*_ = 0.56) presented excessive excitability that resulted in either too large a spontaneous rate (SR > 30 spikes/sec) or insufficient output synchrony (VS < 0.9 or EI < 0.9) due to multiple spiking in one tonal cycle.

As noted above, the On_L_ response pattern is another representative category for GBCs. Some of our model instances presented On_L_-type PSTHs (Fig 6F and 6G) that had a lower sustained rate than PL_N_ units. Parameter distributions of On_L_ instances are shown in Fig 7G–7L. In comparison to PL_N_ units, the number of excitatory inputs of On_L_ instances was biased to large values (Fig 7G) while the excitatory input amplitude was smaller (Fig 7I), suggesting that having many small inputs leads to a low sustained rate. The peak count for the refractory period was slightly shifted to a smaller value (Fig 7J). The other three parameters (Fig 7H, 7K and 7L) had largely similar distributions between PL_N_ and On_L_.

In the 6-dimensional parameter space, the distribution of the accepted model instances may form either a connected or disconnected set. As seen in the image with sparse blue dots (Fig 5E), however, the dimensional stack does not provide the information about the connectivity of the parameter set [68]. And each histogram in Fig 7 shows only one-dimensional projection of the parameter distribution in the 6-dimensional space. Therefore, we performed an additional analysis to test the connectivity of the PL_N_ model instances. Using the most conservative definition of neighbors (i.e., accepting only one-dimensional neighbors; see "Connectivity" in Materials and Methods), we found 104 clusters of instances. Accepting up to three-dimensional neighbors in the grid space, however, resulted in only one large cluster in which all the model instances belonged. This means that each PL_N_ instance can be translated into another PL_N_ instance by changing at most three parameters to their neighboring values in the parameter grid (Table 1). These results suggest that the PL_N_ instances form a largely connected set in the 6-dimensional parameter space. In other words, it is unlikely that the category of PL_N_ units originates from two or more distinct parameter regions.

Depending on the shape of the parameter set in the high-dimensional space, the mean of two accepted models may or may not be an accepted model [42]. To test this with our PL_N_ population, we performed a convexity analysis. We randomly selected two PL_N_ model instances (called *parents*) and checked the response characteristics of *child* model instances created by an interpolation of parameters between these parents. Out of 10500 child model instances tested, 6527 (62.2%) satisfied our PL_N_ criteria. This means that the set of PL_N_ instances is not convex but skewed in the 6-dimentional parameter space. Corresponding to this, we note that the median model, whose parameters were set as the median value of all instances identified as PL_N_ (*M*_*E*_ = 25, *W*_*E*_ = 0.24 ms, *A*_*E*_ = 0.44, *T*_*R*_ = 1.20 ms, *T*_*A*_ = 0.25 ms, *S*_*A*_ = 0.80), was not an acceptable PL_N_ unit, because its spontaneous rate was too high (51.5 spikes/sec). When restricted to the PL_N_ population with one fixed number of inputs *M*_*E*_, however, the median model was still a PL_N_ instance (e.g., Figs 2–4 show the responses of the median model for *M*_*E*_ = 20 inputs). These results agree with previous findings (reviewed in [42]) that nonlinear dependence among parameters should be considered when multiple parameter sets are combined to create a new model instance.

#### Inter-parameter dependence and input-output relations

**Fig 8:**
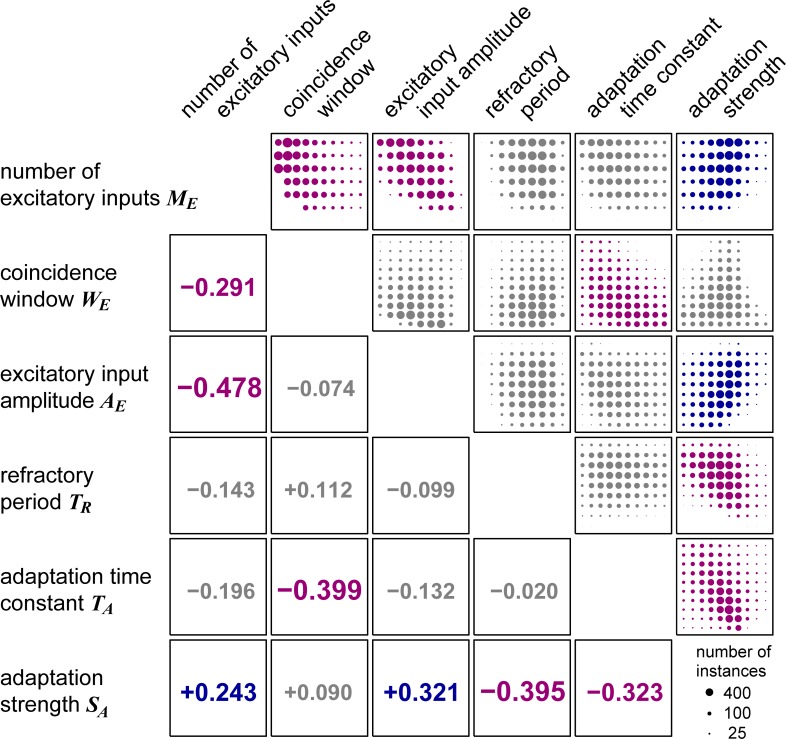
Distribution and correlations of pairs of parameters for accepted PL_N_- and On_L_-type GBC model instances. (**Upper triangle**) Distribution of parameter pairs. Each square panel presents the distribution of a specific parameter combination. The vertical or horizontal axis of each panel spans the range of a corresponding parameter (Table 1). Ascending parameter values are arranged from down to up (along the vertical axis) or from left to right (along the horizontal axis). The area of each filled circle shows the count of accepted parameter pairs. (**Lower triangle**) Correlation coefficients of parameter pairs. Distributions and values are marked in blue (positive correlation) or purple (negative correlation) for parameter pairs with an absolute correlation coefficient over 0.2 (and with p < 10^−9^ for 7520 + 4094 = 11614 instances), and in gray otherwise (i.e., with absolute correlation coefficients below 0.2).

Because of the constraints imposed on the output characteristics, model parameters of the accepted GBC instances cannot vary independently from each other. Fig 8 (upper triangle) illustrates how pairs of parameters for the GBC population covary. Our correlation analysis (lower triangle of Fig 8) confirmed that some parameters should be varied together to keep the GBC-type responses. For example, increase of the number of inputs *M*_*E*_, which normally leads to increased spontaneous and driven rates, can be partly compensated either by reducing the input amplitude *A*_*E*_ or the coincidence window *W*_*E*_ or by increasing the adaptation strength *S*_*A*_. Similarly, long-lasting adaptive effects due to a large *T*_*A*_ can be counteracted in part by either reducing the duration of input *W*_*E*_ or the strength of adaptation *S*_*A*_. Moreover, increase in refractory period *T*_*R*_ has, to some extent, comparable effects to strengthening the adaptation *S*_*A*_, because these two factors are both relevant to the control of interspike intervals through refractoriness. These correlations suggest that some of the model parameters could be combined to reduce the total number of parameters. However, we did not pursue this option, because the observed correlations were rather weak (with absolute coefficients all below 0.5), the number of model parameters was already small, and further reduction of parameters would make the interpretation of individual parameters difficult.

**Fig 9:**
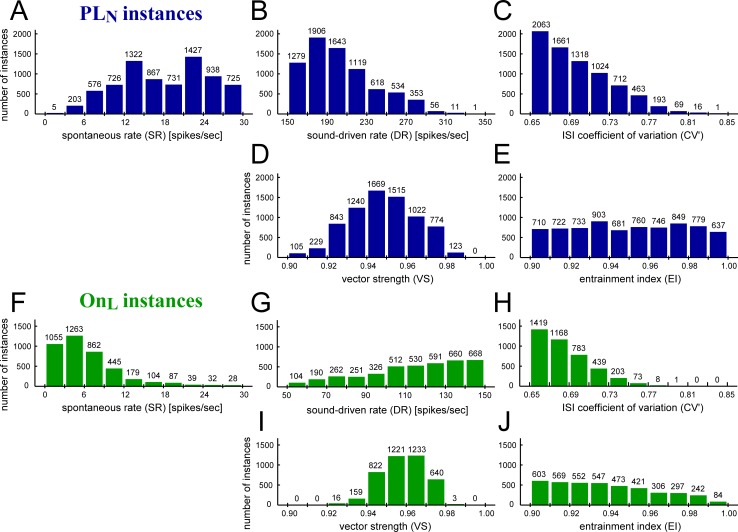
Distribution of output measures for accepted GBC model instances. **A-E:** PL_N_-type instances. **F-J:** On_L_-type instances. **A,F:** Spontaneous rate (SR). **B-C,G-H:** High-frequency tone-driven responses (7000 Hz, 70 dB SPL): steady-state rate (DR: **B and G**) and modified coefficient of variation of ISIs (CV': **C and H**). **D-E,I-J**: Low-frequency tone-driven responses (350 Hz, 70 dB SPL): vector strength (VS: **D and I**) and entrainment (EI: **E and J**). The accepted ranges were: 0–30 spikes/sec (A,F); >150 spikes/sec (B) or 50–150 spike/sec (G); 0.65–0.95 (C,H); >0.9 (D,E,I,J). The total number of model instances in each plot is 7520 for PL_N_ and 4094 for On_L_.

Fig 9A–9E shows the distribution of the output measures of the PL_N_ model instances. Most of our accepted PL_N_ instances had a spontaneous rate of 10–30 spikes/sec and a high-frequency (7000 Hz, 70 dB SPL) sound-driven rate of 150–250 spikes/sec. These ranges roughly correspond to the observed rates in GBCs [22,35,38], except that very low spontaneous rates can be better simulated by On_L_ instances (see below). The distribution of the regularity measure were skewed to the lower end (CV'~0.65; Fig 9C), since the convergence of many subthreshold inputs usually leads to regular outputs [30]. For a low-frequency tonal stimulus of 350 Hz, the degree of phase-locking showed a unimodal distribution centered on 0.95 (Fig 9D), while the degree of entrainment was rather uniformly distributed between 0.9 and 1.0 (Fig 9E). This might indicate that the condition for good entrainment (EI = ~ 0.95) that requires roughly one spike per cycle is more stringent than that for good phase-locking (VS = ~0.95) that can be obtained with a wider range of spiking rates. On the other hand, it is more difficult to achieve perfect phase-locking (VS = ~1.0) than perfect entrainment (EI = ~1.0), because the calculation of EI is not affected by minute variations of spike timings as long as they are within the certain ISI range.

To compare with PL_N_ instances, Fig 9F–9J shows the distributions of the output measures for the accepted OnL instances. The spontaneous rates of most On_L_ instances were less than 10 spikes/sec (Fig 9F), filling the lowest range that were not fully covered by the PL_N_ population (Fig 9A). High-frequency sound-driven rates were skewed towards 100–150 spikes/sec (Fig 9G), because instances with low excitability generally showed insufficient entrainment to low-frequency stimulation and were thus excluded. Regularity of spiking was similar between On_L_ (Fig 9H) and PL_N_ (Fig 9C) instances. Vector strength was, on average, slightly higher for On_L_ (Fig 9I) than PL_N_ (Fig 9D), because On_L_ instances were less likely to have multiple spikes in one period, which would lower the resulting VS. In contrast, entrainment index was skewed to values near 0.90 (Fig 9J), because of the spiking rates of On_L_ instances were often not high enough to reach perfect entrainment.

**Fig 10:**
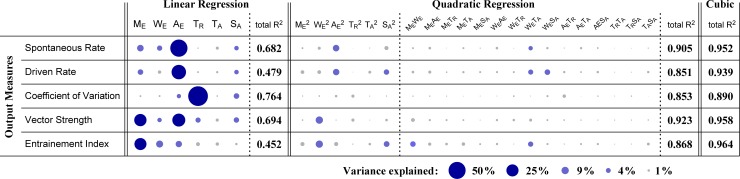
Polynomial fit of model parameters to output measures in accepted PL_N_- and On_L_-type GBC model instances. R^2^-values for the linear, quadratic, and cubic regressions are displayed. For the linear and quadratic fits, relative contribution of each parameter (or each parameter pair) is shown by the area of a filled circle. Circles are colored dark blue for 12% or more variance explained, light blue for over 3% of variance explained, or gray otherwise. *M*_*E*_, number of inputs; *W*_*E*_, coincidence window; *A*_*E*_, excitatory input amplitude; *T*_*R*_, refractory period; *T*_*A*_, adaptation time constant; *S*_*A*_, adaptation strength.

Next, we investigated the relationship between the input parameters (Fig 7) and output measures (Fig 9), using polynomial fits (Fig 10). The total R^2^-values indicate that linear fits were not a good predictor of the output measures (R^2^ < 0.70), except for the coefficient of variation (R^2^ > 0.75). Quadratic fits obtained much better performances (R^2^ > 0.85) than linear fits, and cubic fits provided even better fits for all the five output measures (R^2^ > 0.89). These results imply that not only single parameters but also their nonlinear interactions are responsible for determining the output properties of the model (see below). This is because the generation of spike output requires a nonlinear process of coincidence detection, where simple summation of inputs over time is not sufficient (Fig 1E).

To reveal the contribution of each parameter (and each parameter combination) in the polynomial fits, more detailed analyses were performed (see "Input-output regressions" in Materials and Methods). The size of each filled circle in Fig 10 represents the relative contribution of each linear or quadratic component. We found that the coefficient of variation can be explained mostly by the refractory period *T*_*R*_, indicating that a long refractory period leads to regular spiking. The temporal properties, VS and EI, were explained most effectively by the number of inputs *M*_*E*_ and the input amplitude *A*_*E*_, but were affected also by other factors including the input duration *W*_*E*_ and its square *W*_*E*_^2^. The adaptation strength *S*_*A*_ affected, to some extent, both high- and low-frequency sound-driven responses. Some combinations of parameters showed non-negligible contributions to outputs (Fig 10, quadratic terms shown with light blue circles). For example, several quadratic terms of the input duration (*W*_*E*_^2^, *M*_*E*_*W*_*E*_, *W*_*E*_*T*_*A*_, *W*_*E*_*S*_*A*_) had more than 4% of the contribution (light blue circles) to the observed variances. The spontaneous and sound-driven rates were affected by a number of linear and quadratic factors, supporting our interpretation that a nonlinear interaction plays a role in determining these outputs. Overall, these observations suggest that multiple factors are responsible for determining each of the output measures in our minimalistic model of temporal processing (see [41] for a similar conclusion for more complex conductance-based models). And these interparameter dependences may underlie the non-convexity of the parameter set of the GBC population in the six-dimensional space described above.

### GBC population—Model validation

#### Phase-locking and entrainment

To examine the plausibility of our GBC model population, we tested their temporal response properties under several stimulus conditions that were not used for selecting model instances. Specifically, we used pure tones, high-intensity low-frequency tones, and SAM tones, to confirm the validity of the model instances in a frequency region wider than tested at the model selection stage. Sections below correspond to each of these stimuli.

**Fig 11:**
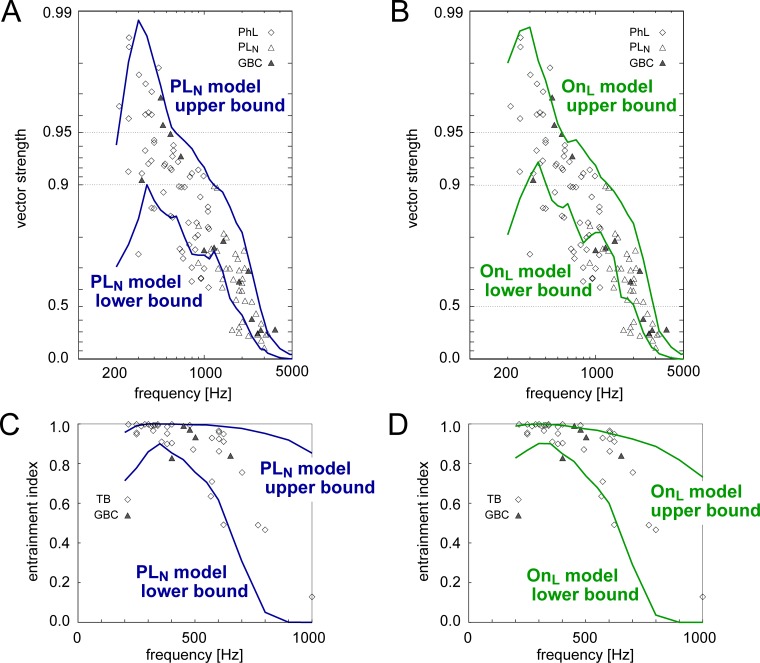
Frequency-dependent temporal properties of the GBC population. **A-B:** Vector strength (VS) of PL_N_ (**A**) and On_L_ (**B**) population plotted along the scale of log(1-VS). **C-D**: Entrainment index (EI) of PL_N_ (**C**) and On_L_ (**D**) population. Solid curves in each panels show the lower and upper bounds of VS and EI calculated for the modeled GBC population (7520 PL_N_-type or 4094 On_L_-type instances). Symbols in A-D show empirical data [25] (see legend of Fig 4 for the description of these data points).

First, we used pure tones at 200–5000 Hz [25] to drive the model PL_N_ instances receiving inputs from AN fibers that have the same characteristic frequency (CF). The simulated upper and lower bounds of VS (Fig 11A) covered the majority of empirical data points obtained from putative GBCs in cats [25]. The entire trend of the simulated VS above 350 Hz followed the frequency-dependent decay of phase-locking. Our On_L_ model instances (Fig 11B) showed generally similar frequency dependence as PL_N_ instances. The population VS at 200–250 Hz for the PL_N_ instances (Fig 11A) was lower than that at 300–350 Hz, because of multiple spikes occurring in one cycle (i.e., so-called peak splitting). This effect was weaker for OnL instances (Fig 11B) due to their lower excitability, which suppressed the generation of multiple spikes in each stimulus cycle.

The distribution of simulated entrainment indices largely matched the reference data in cats (Fig 11C for PL_N_, 11D for On_L_) [25]. Below 500 Hz, EI was mostly between 0.8 and 1.0, and both empirical and simulated distributions became broader at higher frequencies. Similarly to VS, the population EI for the PL_N_ instances at 200–250 Hz was lower than that at 300–350 Hz, again due to peak splitting (Fig 11C), while the On_L_ population showed smaller changes in EI at these frequencies (Fig 11D). Furthermore, the low excitability of On_L_ instances limited the highest possible spike rates, leading to a reduced upper bound of EI for frequencies over 500 Hz (Fig 11D). In the empirical distributions of VS (Fig 11A and 11B) and EI (Fig 11C and 11D), a small number of data points fell below the lower bounds of the simulated values. These data points could be covered if we loosened the selection criteria of VS > 0.9 and/or EI > 0.9 at 350 Hz.

#### Tail-sync responses

**Fig 12:**
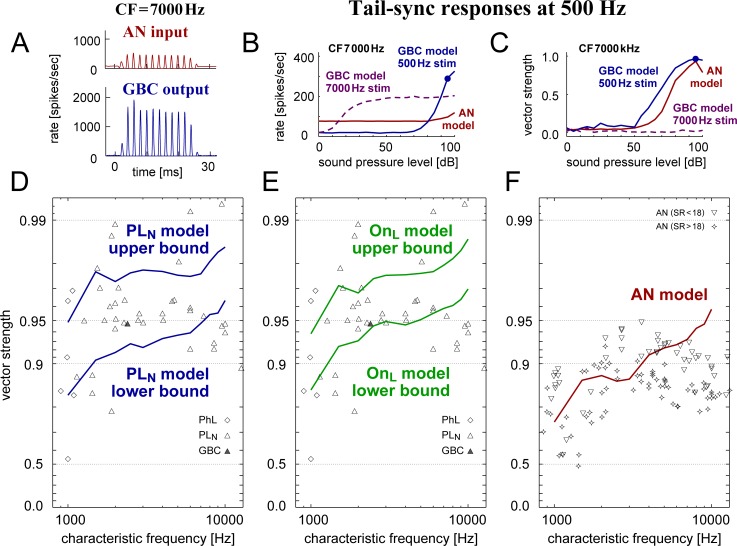
Tail-sync responses. **A:** PSTHs of the AN (top, red) and GBC (bottom, blue) models driven by 500-Hz tone at 95 dB SPL (see Table 1 for the parameter values used). **B-C**: Level-dependent spiking rates (**B**) and phase-locking (**C**) of the same AN (red) and GBC (blue) models as in **A**. GBC responses to CF-tonal stimulation (dashed purple) are also shown for comparison. In **A**-**C**, the characteristic frequency (CF) of the models is 7000 Hz. Filled circles in **B-C** indicate the responses with which the model was further tested. **D-F:** CF-dependent tail-sync phase-locking of the PL_N_ model population (**D:** blue curves), On_L_ model population (**E**, green curves), and the AN model (**F:** red curve) to 500-Hz tone at 95 dB SPL. Symbols in **D** and **E** show phase-lockers (PhL, diamond: most likely to be SBCs or GBCs but possibly other cell types), PL_N_ units (unfilled triangle: presumed to be GBCs), and histologically confirmed GBCs (filled triangle), reported in [63]. Data points in **F** show empirical data of cat AN fibers [63]; different symbols correspond to different spontaneous rate (SR) categories.

High-CF AN fibers and GBCs can phase-lock to low-frequency tonal stimuli, when the sound intensity is sufficiently high [63]. We tested this "tail-sync" property with our PL_N_ and On_L_ population. The model GBC instance (CF = 1–10 kHz) was assumed to receive inputs from AN fibers tuned to the same CF, and the stimulus frequency was fixed at 500 Hz. As shown in previous experiments [63], high-CF units that normally show PL- or PL_N_-shaped PSTHs at high frequencies (7000 Hz; Fig 2A and 2B) lock to the low-frequency tone, presenting an improvement of phase-locking from AN to GBC (Fig 12A). The rate-threshold at 500 Hz (Fig 12B, blue) was several tens of dB higher than the threshold at CF (Fig 12B, dashed purple). At high intensities, phase-locking was much more prominent for the low-frequency stimulus (Fig 12C, blue) than for the CF tone (Fig 12C, dashed purple). These results matched previous *in vivo* observations [63].

We tested the low-frequency tail-sync phase-locking of our GBC model population. The simulated VS range matched the empirical median VS value of 0.95, covering about a half of the empirical data points in a CF range of 1000–10000 Hz (Fig 12D for PL_N_, 12E for On_L_). The distribution of data points, however, was broader in experiments [63] than in our simulation results. Both the AN and GBC stages can be responsible for this discrepancy. Empirical AN tail-sync phase-locking showed large variations (data points in Fig 12F), while the AN model we used did not have such variability and its VS almost monotonically increased with CF (Fig 12F, red curve). Furthermore, we used the same GBC model population for different CFs, although the real GBCs may have tonotopic variations of cellular and synaptic properties that could affect the temporal fidelity of spike generation. We will revisit this point in Discussion.

#### SAM responses

**Fig 13:**
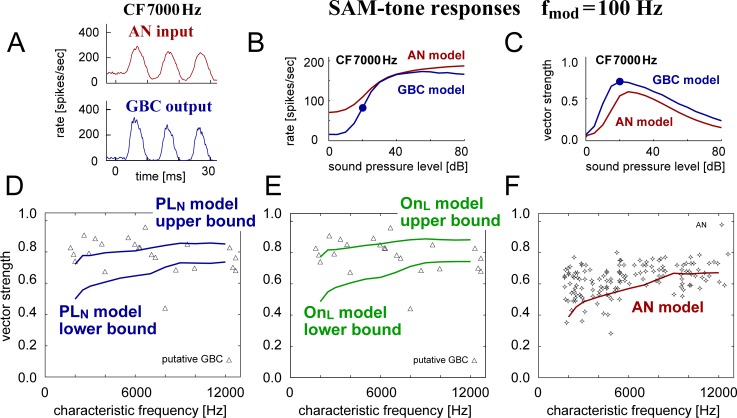
SAM-tone responses. **A:** PSTHs of the AN (top, red) and GBC (bottom, blue) models driven by 100-Hz SAM tone at 20 dB SPL (see Table 1 for the parameter values used). Characteristic frequency (CF) of the models is 7000 Hz. **B-C:** Level-dependent spiking rates (**B**) and envelope phase-locking (**C**) of the same AN (red) and GBC (blue) models as in **A**. Filled circles in **B-C** indicate the condition for which the model showed the maximum VS. **D-F:** CF-dependent envelope phase-locking of the PL_N_ model population (**D:** blue curves), On_L_ model population (**E:** green curves), and the AN model (**F:** red curve) to 100-Hz SAM tone at 20 dB SPL. In **D-E**, the maximum VS was computed for sound intensities between 10 and 45 dB SPL (5 dB steps). Triangles in **D** and **E** indicate empirical single-unit data of presumed GBCs, and hollow diamonds in **F** show AN fiber data recorded in cats [66].

High-CF auditory neurons including AN fibers and GBCs can also phase-lock to the envelope of amplitude-modulated sounds. We examined this envelope phase-locking with our PL_N_ and On_L_ instances (CF = 2–12 kHz). The model CF matched the carrier frequency of the SAM sound. GBCs showed an enhanced phase-locking to the stimulus envelope compared to AN inputs (Fig 13A). While the simulated spike rates almost monotonically increased with the sound level (Fig 13B), VS showed a non-monotonic level dependence for both AN and GBC (Fig 13C), agreeing with empirical observations [66,69]. The highest VS was achieved at the point around which the rate-level curve had a maximal slope (Fig 13B and 13C, filled blue circles). The decrease of VS at high levels is due to multiple spike occurrence in one cycle (i.e., the neuron fired at up to 200 spikes/sec, whereas the envelope frequency was 100 Hz), which fills the trough portion of the phase histogram [69].

For each instance, we varied the sound pressure level and used the level at which the output VS becomes maximal. The simulated range for the maximal VS (Fig 13D for PL_N_, 13E for On_L_) overlapped some of experimental data recorded from putative GBC units in cats [66]. The empirical median VS value of ~0.8, however, was located near the upper bound of the simulated VS. Moreover, many empirical data points, especially units with CFs below 7000 Hz, had even higher VS values than the upper bound of the model instances. This discrepancy could be explained, at least partly, by the relatively poor envelope phase-locking of the AN model compared to the actual AN data in cats (Fig 13F). Up to about 7000 Hz, the simulated VS of the AN model (Fig 13F, red curve) was close to the lower limit of the empirical data (symbols in Fig 13F).

#### Frequency tuning

**Fig 14:**
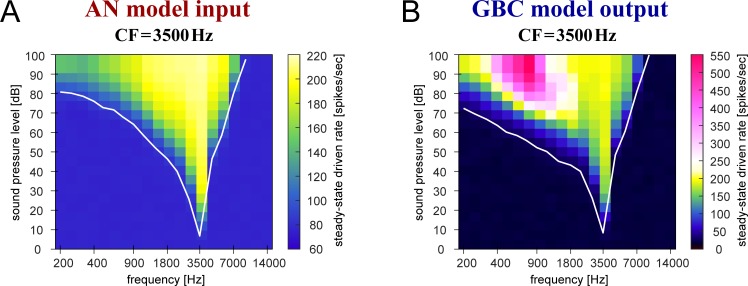
Simulated frequency response areas (FRAs). **A:** FRA of the AN model. **B:** FRA of the default GBC model (see Table 1 for the parameter values used). CF of both models was fixed to 3500 Hz. The level and frequency of the stimulus tone were varied and the resulting spiking rates of the models were calculated and plotted in color. In both panels, the same color code was used for the range of 60–220 (spikes/sec) to facilitate comparisons. White curves show the estimated rate-level threshold at each frequency, defined as the sound pressure level at which the spiking rate was 10 (spike/sec) higher than the spontaneous rate.

The frequency selectivity of an auditory neuron can be assessed with its frequency response area (FRA). Fig 14 shows simulated FRAs of the AN model and the default GBC model instance. Each color-coded pixel in the FRA plot represents the spiking rate at one specific frequency and level. Both panels in Fig 14 have bright areas (yellow to pink) at similar locations, indicating that the frequency-tuning of the GBC model is largely inherited from the frequency-tuning of the AN model. The highest spiking rate of the GBC model, however, was observed at a frequency below 1000 Hz (Fig 14B, dark pink area), while the highest rate of the AN model is attained at the modeled CF of 3500 Hz (Fig 14A, light yellow area). The pronounced entrainment capability of the GBC (exemplified in Figs 4A and 4B, 11C and 11D), in combination with low-frequency phase-locking of converging AN fibers, is responsible for this marked increase of GBC spiking rate. Such prominent spiking activity to high-level, low-frequency tones are a common feature of GBCs [24,27,35].

At the high-frequency edge of the FRA, a considerable fraction of GBCs recorded *in vivo* showed a reduction of spiking activity below the spontaneous rate, which is called sideband inhibition or lateral suppression (cats: [23,24,70]; gerbils: [38]; see Discussion). However, since our present GBC model framework lacks inhibitory inputs, the FRA of the simulated GBC does not have an inhibitory sideband (Fig 14B). Broadband inhibition (putatively from D-stellate cells [54]) would be necessary to simulate such sideband effects in GBCs.

The white V-shaped curve in Fig 14A shows the threshold sound pressure level of the model AN at each stimulus frequency. This frequency-tuning property is well preserved between the AN and GBC (Fig 14B), especially near and above the modeled CF. At low frequencies (< 1.5 kHz), however, the GBC model had lower thresholds than the AN model, because the AN input can phase-lock at sound levels even below the rate threshold [25,63] (see Fig 3A and 3B or Fig 12B and 12C). The steepness of a frequency-tuning curve can be quantified with a Q_10_ value, which is defined as the CF divided by the bandwidth of the tuning curve measured at 10 dB above threshold. Previous *in vivo* recordings in cats reported that the Q_10_ value generally increases with CF in both ANs [71,72] and GBCs [35,62], and the distribution of the Q_10_ values greatly overlap between ANs and GBCs. According to these studies, Q_10_ values of a large majority of ANs and GBCs with a CF of 2000–6000 Hz were in the range of 2–8 and clustered around 4–6. The Q_10_ values of our models (CF = 3500 Hz) calculated from their tuning curves were 5.7 (AN: Fig 14A; see [58]) and 5.5 (GBC: Fig 14B), both matching empirical data. FRAs were not used for further validation of our entire GBC model population, because their calculation requires large computational costs especially for varied CFs, whereas the resulting frequency tuning of each GBC model seemed to follow the tuning of input AN fibers.

### Computational time

In order to assess the computational efficiency of the AN and GBC model used in this study, we measured the computational time spent for the calculation of a 500-second long spiking response (Table 3). Computational time of the AN model depended strongly on the CF of the modeled AN fiber and on the duration of each trial, even though the total length of the simulated time was the same. Computational time of the GBC model did not depend on these factors. The GBC model was much faster than the AN model with the approximate algorithm for its power-law adaptation (Table 3, three columns on right). It should be noted that the actual time spent for the AN stage will be multiplied by the number of AN fibers used for driving one GBC. Therefore the amount of computational time for simulating the response of one GBC instance is indeed spent mostly by the AN stage. In sum, the simple GBC model introduced here does not make a computational bottleneck of the entire simulation. The computational efficacy of the model is further supported by our previous comparison of different models, which revealed that the shot-noise models similar to our GBC model were 10–100 times faster than conductance-based models that require solving differential equations at each time step [55].

**Table 3:** Computational time required for calculating 500-sec responses. "AN exact" and "AN approx" refer, respectively, to the exact and approximate calculation of the power law adaptation of the modeled AN (see [59]). Computations were carried out with Matlab 2018a (MathWorks) on a desktop computer (Dell Precision T1700) with 64 bit Windows 7 Professional Operating System, Intel Xeon CPU E3-1270 v3 (4 core, 3.5 GHz) and a 16 GB memory.

	AN exact 350 Hz CF	AN exact 7000 Hz CF	AN approx 350 Hz CF	AN approx 7000 Hz CF	GBC model
50-ms tone at CF 10000 repetitions	869.6 sec	77.0 sec	65.0 sec	65.0 sec	1.22 sec
5000-ms tone at CF 100 repetitions	776.6 sec	801.6 sec	20.0 sec	28.9 sec	

## Discussion

### Model GBC population and biophysical substrates

In this study, we created a population of simple models and replicated known physiological output features of GBCs. Employing a model with a small number of parameters enabled us to simulate neuron-to-neuron variability of GBCs in a simplified setting, which we expect to complement computational studies using biophysically detailed models. The parameters of the model were systematically varied and each model instance was tested for its spontaneous rate, low-frequency sound-driven temporal spiking patterns (VS and EI), and high-frequency sound-driven rate, regularity (CV'), and PSTH. The population of resulting 7520 PL_N_ and 4094 On_L_ model instances (out of 567,000) presented reasonable fitting to empirical frequency-dependent distributions of phase-locking and entrainment (Figs 11–13). We assume that simulating the variability of GBC responses is important, because neuronal heterogeneity is likely to contribute to an improvement of the overall performance of population coding [73].

Our systematic examinations of the large number of parameter combinations were enabled by the simple structure (Fig 1E) and corresponding computational efficiency of the model (Table 3). Prior modeling studies used similar-sized (~0.6 million [41]) or even larger (~1.7 million [39]) populations to examine how each parameter contributes to a rhythmic behavior of a conductance-based model. Their model was much more complex than our coincidence detection model. In our study, however, we had to repeat the same stimulation up to 1000 times to calculate the stable model outcome (rate, VS, PSTH, etc.), while their simulations required only one trial per parameter set.

Simple models can complement more complex models by shedding light on the fundamental properties of the system under study [43,55,74,75]. The adaptive coincidence counting model we used in this study aimed to replicate the input-output relations of the GBC without considering its biophysical details. A similar shot-noise type model with an exponential postsynaptic response was used in a previous modeling study [48], which found that a small number of parameters (five out of eight in their study) determined the PSTH shapes of modeled cochlear nucleus neurons. In a complex biophysical model, a similar activity pattern can often be seen in distinct regions of its parameter space, because many physiological properties are affected by multiple factors and some parameters can compensate with each other [40,76–79]. Such correlations of parameters, sometimes called parameter tradeoff, were also seen in our simplified model (Fig 8).

The adaptive threshold of our model was found essential to replicate the PL_N_/On_L_-type response; weakening or removing the threshold adaptation led to chopper-type PSTHs (Fig 6H) that are not typical of GBCs. A computational study using an adaptive integrate-and-fire model [50] demonstrated the importance of threshold adaptation in precisely replicating the temporal spiking patterns of bushy cells. Our analyses using polynomial fitting showed that the adaptive threshold is involved in controlling the output spiking rates and phase-locking (Fig 10). The degree of phase-locking (VS) was improved with an appropriate amount of adaptation that suppresses the generation of more than one spike per period.

Activity-dependent threshold regulations in auditory neurons are mediated by multiple biophysical factors, including the activation of low-voltage-activated potassium (KLVA) channels, inactivation of sodium channels, synaptic depression and inhibition (e.g., [30,50,80–83]). For example, KLVA channels, which are opened by slight depolarization of the membrane potential, counteract the accumulation of synaptic inputs and prevent asynchronous inputs from reaching the threshold [30,52]. A previous model of bushy cells [50] used threshold adaptation triggered by output spike generation. In our model, spike-triggered effects were summarized into the absolute refractory period, and the dynamic threshold depended only on the input history but not on output spikes. Moreover, our model has only one adaptation time constant, although the underlying mechanisms may include multiple time scales. Further refinement of the model might require the consideration of both sub- and suprathreshold adaptations.

The length of the coincidence window is determined by several factors, such as the membrane and synaptic time constants (see [57] for more detailed discussion). Our population of models calibrated with available cats' data had coincidence time windows mostly between 0.1 and 0.5 ms (Fig 7B and 7H). This range is slightly lower than the input time scale of ~0.5 ms in rodent bushy cells measured *in vitro* (mice [84,85]; rats [86]) and *in vivo* (rats [87]), possibly reflecting the difference between cats and rodents. The optimal lengths of the refractory period of our model centered on 0.9–1.4 ms (Fig 7D and 7J), roughly comparable to the estimated refractory period of 1.4 ± 0.4 ms [50]. As already pointed out in Results, we note that the output characteristics of the model are generally determined not by a single parameter but by nonlinear interactions of multiple model parameters (Fig 10) [41], which is in line with the non-convex shape of the parameter set of the modeled GBC population [42].

### Assumptions and limitations of the model

Our GBC model naively assumed that the amplitudes of excitatory inputs do not vary between input fibers. In our modeled GBC population, the input amplitude was distributed around 0.4 for the accepted PL_N_ units (Fig 7C) and 0.3 for the On_L_ units (Fig 7I), indicating that at least 3–4 coincident inputs were required for eliciting a spike (with the static threshold fixed to 1.0). This observation is consistent with previous theoretical results showing that convergence of multiple AN fibers contributes to the improvement of phase-locking [30–34]. Anatomical investigations found that the sizes of synapses can vary not only across GBCs but also within one GBC [7,10,11,13,88]. Furthermore, some of the excitatory inputs to GBC may even be suprathreshold [24]. In addition, bushy cells receive synaptic inputs of noncochlear origin and form gap junctions with neighboring bushy cells [88]. Possible effects of having such heterogeneous synapses on temporal coding would be a topic of a future computational study.

Convergence of multiple inputs generally leads to a pronounced PSTH peak at the onset (as shown in Fig 6), with a reduced jitter in the first spike latency [30,47]. This jitter reduction between AN inputs and PL_N_ outputs was observed in previous *in vivo* recording studies [22,35,36,89]. Because of the rectangular input shape (Fig 1E), the output timing of the GBC model always coincides with the timing of one of its AN inputs. In other words, our GBC model does not account for the axonal and/or synaptic delay between ANs and GBC. As a first-order approximation, this discrepancy could be partly compensated by introducing a fixed delay of 0.1–0.5 ms. Such an issue of spike-timing might become more prominent with inputs that are only slightly above threshold. Conductance-based models [30,50] showed that the spike generation can be delayed for barely suprathreshold inputs. To account for this delayed spiking with our model, the rectangular input would need to be revised with a more biologically realistic input shape.

The input AN fibers in our model was assumed to share the same CF. This assumption of narrowband tuning is supported both by the tonotopic projections of AN fibers (reviewed in [5]) and by the narrow frequency tuning curves of bushy cells comparable to those of AN fibers [35,61] (see Fig 14). In addition, we assumed that our GBC model received inputs only from high-SR AN fibers, because a prior anatomical study estimated that the distributions of AN fibers projecting to GBCs are biased to high-SR units [7]. Adding variability to CF and/or SR of input fibers would reduce tone-driven responses of the GBC model, because the activity of off-CF or medium/low-SR fibers will be lower than that of on-CF, high-SR AN fibers. A previous modeling study [53] noted that the tone-driven firing properties of a modeled GBC were not significantly affected by the inclusion of a small number of medium- or low-SR fibers. For SAM tones, however, lowering the SR of some input fibers might still contribute to the improvement of phase-locking in GBCs, as medium- and low-SR AN fibers generally show better envelope phase-locking than high-SR fibers [69].

In the present study, we considered only excitatory AN inputs to GBCs, to keep the model as simple as possible. Because of this simplification, the modeled GBC lacks inhibitory sidebands that would appear at the high-level, high-frequency portion of the FRA (Fig 14B). A number of prior anatomical studies, however, found inhibitory projections to bushy cells [10,13,88,90,91]. Sources of inhibition include D-stellate cells in the AVCN with a broad frequency tuning [91] and tuberculoventral (vertical) cells in the dorsal cochlear nucleus having a narrow frequency tuning [91,92]. Commissural multipolar cells in the contralateral cochlear nucleus can also be an additional source of inhibition [93,94]. In response to monaural on-CF tonal stimulation, a majority of GBCs show monotonic rate-level functions [12,95]. Hence the role of on-CF inhibition was simply assumed to be gain control [62,96] and/or echo suppression [97]. In addition, roughly half of GBCs recorded *in vivo* have inhibitory sidebands in their FRAs [23,38,70,98], which may contribute to the sharpening of frequency selectivity. Because of their low spontaneous spiking rates, however, sideband inhibition in GBCs is not always apparent and has rarely been investigated systematically [24], making a contrast to inhibition in SBCs with high spontaneous rates that has been studied extensively in the last decade [99–103].

Possible benefits of lateral inhibition will probably be more apparent with complex sound stimuli that are rich in spectral components, since our results suggested that responses to on-CF tones can be reliably simulated without inhibition (Figs 4 and 11). Furthermore, many cochlear nucleus neurons are inhibited by contralateral sound stimuli [104–107]. Measured frequency-tuning and latency of commissural inhibition showed a large variation, suggesting that both direct and indirect projections (and hence a variety of functions) may be associated with this contralateral inhibition [106]. Further experimental and theoretical studies are required to fully understand the physiological roles of these inhibitory inputs to GBCs. A recently developed model framework that simulates ipsilateral inhibitory connections from D-stellate cells and tuberculoventral cells to bushy cells [54] might be useful towards this end.

In our model selection, we drove each model instance with both high- and low-frequency tones, under the assumption that the membrane properties of GBCs are uniform along the tonotopic axis. This assumption could be a reason for the discrepancy between modeled responses and empirical data for tail and SAM tones (Figs 12D and 12E, 13D and 13E). Previous experimental studies reported tonotopic variations of ionic conductances in AN [108], LSO [109], MSO [110] and MNTB [111], as well as tonotopic regulations of bushy cell development [112] in several rodent species. In mice bushy cells, however, expressions of potassium channels were rather uniform along the frequency axis [82]. Overall, it is still largely uncovered what kind of tonotopic variations in bushy cells exist and how they might affect the temporal spiking patterns.

For tonal stimulation, the simulated spike rate, phase-locking, and PSTH shape of the GBC model were stable for sound levels over 60 dB SPL (Fig 3). This response stability enabled us to use a fixed tone intensity of 70 dB SPL in our selection of models (Figs 5 and 6) and in the calculation of population phase-locking at the CF of the model (Fig 11).These results do not guarantee, however, that responses are level-invariant for other sorts of sound stimulation. For example, envelope phase-locking to SAM tones depends non-monotonically on the sound level (Fig 13B and 13C). Spiking responses to off-CF tones can be more strongly affected by the sound level than those to on-CF tones (compare the responses at 600 Hz and 3500 Hz in Fig 14B). Inhibitory inputs discussed above as well as olivocochlear gain control [113] would become more relevant for high-intensity or broadband signals (see "Applications and future directions" below).

### Response criteria and classification of PSTHs

Using the selection criteria for PL_N_/On_L_ units, we aimed to cover most fundamental response characteristics of GBCs. As pointed out before [38], response properties of bushy and other cells in the AVCN appear to form a continuum in the multidimensional space, rather than making distinct clusters. This is confirmed by the connectivity analysis of our PL_N_ model population. Previous experimental results in cats showed that a large majority of GBCs have an SR of 0–30 spikes/sec, which we adopted for our criterion; but there was actually no clear boundary for the representative SR range of GBCs [15,62]. Instead, the number of recorded GBC units gradually decreased with SR up to 60–100 spikes/sec. Furthermore, our model instances showed a gradual change in SR (and other output measures) with varied parameters. For these reasons, it is highly likely that we missed some instances that might have been classified as GBC-like if different criteria had been used.

The parameter ranges we used (Table 1) might not cover the full spectrum of real GBCs. From the distributions of accepted parameters (Fig 7) and from the connectivity analysis that suggested the existence of one big parameter cluster, we expect that our ranges overlapped the most relevant part of the 6-dimensional parameter space grid. However, a small number of instances located outside of our search grid may still present GBC-like responses. For example, a relatively broad distribution of the adaptation time constant *T*_*A*_ (Fig 7E and 7K) suggest that some instances with *T*_*A*_ > 0.5 (ms) may still satisfy our selection criteria for PL_N_ or On_L_.

A continuum of response properties appears not only in quantitative output measures (including SR and VS) but also in more qualitative ones such as PSTH shapes. As shown in Fig 3D, the PSTH of a GBC can be transformed from PL to PL_N_ depending on the stimulus sound level [19,24,36]. The intermediate PSTH shapes (e.g., Fig 3D, 40 dB SPL) are on the borderline between these two categories. The PSTH of one anatomically confirmed bushy cell appeared to be chopper-like [21], although a chopper PSTH is normally associated with stellate cells in the AVCN [2,22]. Early studies established the categories of PSTH types in the cochlear nuclei [35,36,114,115] and developed a corresponding decision tree [22]. However, there were always a small number of units classified as "unusual", "extraordinary", or "intermediate" [22,36,115–117], reflecting the continuous (and non-exhaustive) nature of the established PSTH categories. Our population modeling approach might be useful for examining what factors contribute to these non-canonical response patterns.

Although GBCs are widely associated with PL_N_ and On_L_ units, the distinction between these two categories has long been a subject of discussion. Classically, units with a sustained discharge rate below 100 [22,36] or 150 (spikes/sec) [30] are classified as an onset-type, but this criterion is nonetheless arbitrary [36]. It is generally difficult to clearly distinguish these two patterns [22,117], and therefore in some studies they were jointly grouped as PL_N_ [38,63]. In this study, we separately analyzed PL_N_ and On_L_ instances, but their populations seemed to be located next to each other. For example, an instance with a high-frequency-tone-driven rate of ~150 (spikes/sec) was located on or near the border of the two categories. Moreover, the conventional category of On_L_ [22] is a mixture of two different patterns: units with low sustained rates and units with a prolonged notch after onset [24]. It is unclear whether and how this second subcategory of On_L_ pattern is relevant to GBCs. Our PSTH example shown in Fig 6I could be related to this type, but we nevertheless excluded it from our GBC population by assuming that such a dip pattern was atypical for bushy cells [30].

### Applications and future directions

By varying the parameters, various types of PSTHs can be produced with our model (Fig 6). Chopping responses (Fig 6H) that are regarded as a signature of stellate cells [2,22] were linked to model instances with no or weak threshold adaptation. This relationship would correspond to the observation that T-stellate cells in the ventral cochlear nucleus that lack KLVA conductance show weak adaptation and chopping PSTHs [2]. Some "pauser" units in the dorsal cochlear nucleus [116,118,119] and in the inferior colliculus [120] have a relatively long silent period (5–20 ms) after the onset peak. This response pattern resembled our dip-type responses (Fig 6I and 6J). A previous modeling approach using a shot-noise model replicated multiple PSTH patterns by changing a small number of parameters [48]. These observations suggest that the adaptive coincidence counting model presented in this study may be applied to a wider range of cochlear nucleus neurons than GBCs. We expect that the simplicity of the model would facilitate future theoretical analyses to reveal how and what factors determine response patterns found in the auditory system.

As noted in the Introduction, both GBCs and SBCs present enhanced phase-locking yet possibly through different mechanisms. SBCs receive only few inputs (1–4 in cats [6,7]) that seem insufficient to achieve pronounced phase-locking and entrainment (reviewed in [29]). In gerbils, many SBCs present sideband inhibition and/or non-monotonic rate-level functions [99–102], and phase-locking of SBCs deteriorates by blocking inhibitory inputs [103]. A modeling approach suggested that temporally precise inhibition in SBCs may efficiently reject weak, poorly timed excitatory inputs and contribute to an improvement of temporal fidelity of output spikes [101]. These results indicate that the application of our model to SBCs may require the introduction of inhibitory inputs, as was done in our previous LSO neuron modeling [57].

In this study, we used only limited variation of sounds to drive the model, such as pure and SAM tones. Previous experimental studies of AVCN neurons have used a wider variety of stimuli, including narrow- or broadband noise [121], rippled noise [122–124], tones in noise [125], tones with forward-masking noise [126], amplitude-modulated tones with two-tone complex envelope [127], Schroeder-phase harmonic complex [128], and synthetic or spoken vowels [27,129–134]. We note that the applicability of our current AN-GBC model framework has not been confirmed with such complex sounds. In order to apply complex stimuli to the GBC model, the AN input stage first needs to be tested and calibrated, since the AN model used in this study [61] showed relatively poor phase-locking to SAM tones for CFs below 7000 Hz (Fig 13F). Furthermore, as noted above, potential effects of (the lack of) inhibition may become more prominent for spectrally rich signals that would activate multiple frequency channels. Future studies combining a revised AN front end with a population of bushy cell models will help us simulate and understand how complex sounds are processed along the auditory pathways to enable acoustic communication and binaural sound localization.

## Materials and methods

### GBC model and sound stimuli

#### Adaptive coincidence counting model

The coincidence counting model, a member of the shot-noise model family [44,55], was formerly used to simulate the temporal coding of MSO [56] and LSO neurons [57]. In this model, the number of synaptic input is counted in a time window of length *W*_*E*_. If this number of inputs reaches the pre-defined threshold, then an output spike is generated. In other words, each synaptic input is converted into a rectangular postsynaptic response of length *W*_*E*_ and amplitude *A*_*E*_, and the summed response *v*(*t*) is compared to the threshold *θ*. After each spike generation, the model does not produce any more spikes for a time duration of *T*_*R*_ that corresponds to the absolute refractory period.

To apply this model to GBCs, we modified it by replacing the constant threshold with an adaptive threshold that temporally varies according to the input history of the model (Fig 1E). The threshold of the model *θ*(*t*) is decomposed into the static and dynamic part *θ*(*t*) = *θ*_*S*_ + *θ*_*D*_(*t*). The static part does not change with time and is fixed to one: *θ*_*S*_ = 1. The dynamic part obeys the first order differential equation:

$$T_{A}\left(d\theta_{D}/dt\right)=‐\theta_{D}\left(t\right)+S_{A}\cdot v\left(t\right),$$

where *T*_*A*_ and *S*_*A*_ are the time constant and the strength of adaptation, respectively. This equation implies that the dynamic threshold develops according to the summed input counts with the time constant *T*_*A*_. Without inputs, the dynamic part of the threshold gradually approaches to zero. Because of this adaptive threshold, only well synchronized inputs can lead to spike generation (compare two cases of three inputs in Fig 1E).

In the numerical implementation of the model, we used a time step of *Δt* = 0.01 ms. Input/output timing of the model is restricted by this time step. Because of the rectangular postsynaptic response shape, the summed response *v*(*t*) is constant within the open interval between any two consecutive time steps *t*_j_ and *t*_j+1_ = *t*_j_+*Δt*. This implies that we do not have to approximate the differential equation, but can simply obtain the exact digital solution at each time step [135]. Namely,

$$\theta_{D}\left(t_{j}+\Delta t\right)=exp\left(‐\Delta t/T_{A}\right)\cdot \theta_{D}\left(t_{j}\right)+\left(1‐exp\left(‐\Delta t/T_{A}\right)\right)\cdot S_{A}\cdot v\left(t_{j}\right).$$

We used this formula to calculate the adaptive threshold in a step-by-step manner.

#### Auditory nerve input model

We simulated the inputs to the GBC using the auditory periphery model developed by Zilany, Bruce and others [58–61]. Specifically, we used their 2018 version for our simulations [61]. An early study in cats showed that a GBC receives excitatory inputs primarily from high spontaneous rate AN fibers [7]. We therefore set the spontaneous rate of the AN model at 70 (spikes/sec) throughout our simulations. For the additional parameters of the model, an absolute refractory period of 450×10^−6^ (sec) and a relative refractory period of 512.5×10^−6^ (sec) were used, which are the mean of their default values specified in the model implementation [61]. In our preliminary simulations, we varied these refractory parameters and found no significant effects on the output properties we examined. The GBC model was assumed to receive *M*_*E*_ auditory nerve inputs that share the same characteristic frequency (CF). Specific CFs of the model used are given in the legend of each figure. Since the GBC model introduced above does not have internal noise sources, the output variability of each fixed GBC model instance is solely due to the stochastic nature of the modeled AN fibers.

#### Sound stimuli

Each trial of CF-tonal and tail-tonal stimulations contained short tone burst of 25 ms with a linear rise/fall of 3.9 ms [25]; we repeated 1000 trials to calculate the output measures of the model (see next sections). For the calculation of sustained response properties, we used the spike responses in the time window between 10 and 25 ms after the stimulus onset [25].

For tonal stimuli, we varied the frequency between 200 and 12000 Hz; the default sound intensity was 70 dB SPL, unless otherwise noted. The CF of the model was set to the frequency of the tone. For tail-tone stimuli, the frequency was fixed to 500 Hz to facilitate comparisons to *in vivo* data [63], while the CF of the model was varied between 1000 and 10000 Hz; the default sound intensity level was 95 dB SPL, unless otherwise noted. In the calculation of a frequency receptive area (FRA), the model CF was fixed to one frequency (3500 Hz in Fig 14) and the frequency and intensity of the tonal stimulus were varied.

For SAM-tonal stimuli, we applied a low-frequency sinusoidal envelope (100% depth) to a 600-ms carrier tone at the CF [136]; we repeated 80 trials to calculate the output properties of the model. The sound intensity was varied between 0 and 80 dB SPL with a step of 5 dB. The modulation frequency was fixed at 100 Hz to enable comparisons with available data [66]. The carrier frequency matching the CF of the model was varied between 2000 and 12000 Hz. The first 10 ms of the model responses (either AN or GBC) were discarded in the calculation of sustained responses [136].

### Output measures

#### Spontaneous and sound-driven rates

Spontaneous responses of the model were obtained without sound stimulus. An admissible GBC model needed to have a spontaneous rate of 0–30 (spikes/sec). Although this upper rate of 30 (spike/sec) is based on previous *in vivo* recording results, we note that a minority of GBCs may actually show higher spontaneous rates [15,38,62]; also see Results and Discussion.

Sound driven spiking rates of the model were calculated from the sustained part of the response (10–25 ms after stimulus onset). In response to a 7000 Hz tone at 70 dB SPL, the sustained driven rate had to be 150 (spike/sec) or higher to be classified as PL_N_, and 50–150 (spikes/sec) as On_L_. Prior studies used the threshold of 100 (spikes/sec) [22,36] or 150 (spikes/sec) [30] to distinguish PL_N_ and ON_L_ units. We adopted the higher value, because the stimulus intensity (70 dB SPL) used was fairly high and we wanted to guarantee that the model should still show a PL_N_-type response at lower intensities.

#### Regularity

Regularity of the model spike output was measured as modified coefficient of variation (CV'). This measure is defined as: CV' = σ_ISI_ / (μ_ISI_ - μ_0_), where μ_ISI_ and σ_ISI_ are the mean and standard deviation of the interspike intervals (ISIs), respectively, and μ_0_ = 0.5 (ms) is the correction factor for the dead time of the response [30]. In response to high-frequency tones, the activity of a GBC is relatively irregular, with observed CV' values scattering between 0.65 and 0.95 [30]. We calculated CV' of the sustained response of each model instance driven by a 7000 Hz tone at 70 dB SPL, and excluded the instances whose CV' was out of this range.

#### Phase-locking and entrainment

Phase-locking to low frequency stimuli (and to low-frequency envelopes of SAM sounds) was measured as vector strength (VS) [137], which is defined as:

$$\mathrm{VS}=(1/N)|\sum_{k=1}^{N}\exp(2\pi ift_{k})|,$$

where *N* is the number of spikes, *f* is the reference frequency, *t*_*k*_ is the timing of the *k*-th spike. The entrainment index (EI) of a spike train is calculated from the interspike intervals [25] as:

EI=(numberofISIsbetween0.5/fand1.5/f)/(totalnumberofISIs).

In some plots, we used a scale of log(1-VS) to extend the area for VS > 0.8 and to allow for a direct comparison with relevant experimental data [25].

According to previous *in vivo* recordings, a majority of GBCs show enhancement of VS and EI compared to AN fibers for frequencies below 700 Hz [25]. Most of our model instances showed the highest VS and EI around 300–400 Hz. We therefore set the criteria of VS > 0.9 and EI > 0.9 for 350 Hz tone at 70 dB SPL to judge if the model instance was regarded as a valid GBC model.

#### PSTH shapes

For each model instance driven by 7000 Hz tone at 70 dB SPL, we calculated the peristimulus time histogram (PSTH). We used a time bin of 0.1 ms [22] and applied five-point triangular smoothing. One of the most prominent response characteristics of GBCs is the primary-like-with-notch (PL_N_) PSTH, in which the spiking activity is maximal at the onset followed by a short silent period of about 1 ms [12,19,22–24]. Model instances we tested always showed the highest spike rate at the onset, reflecting the maximal AN input rates (Fig 2A, right). After the onset peak, however, model PSTHs presented a variety of shapes, including those shown in Fig 6.

To distinguish PL_N_ (and On_L_) PSTHs from other response types, we set the following criteria for the simulated PSTH shape (Fig 1F): (P1) the first notch should be below 90% of the sustained rate; (P2) the width of the first notch (measured at 90% of the sustained rate) should be between 0.15 and 1.5 ms; (P3) the second peak rate should be below the half of the first peak rate; (P4) the second notch should be either non-existent or shorter than 0.85 ms. The criteria (P1) and (P2) ensure a clear notch, while (P3) and (P4) were important for rejecting chopper-type responses (e.g., Fig 6H). In addition, (P2) and (P4) were used for excluding dip-type PSTHs (e.g., Fig 6I and 6J), which have a longer silent period than PL_N_ and are uncommon for bushy cells [30].

To test the validity of the above-described criteria, we visually inspected the PSTHs of about 2000 accepted PL_N_/On_L_ instances and randomly selected another 2000 instances that did not satisfy our GBC criteria. We note that our method reasonably detected PL_N_ and On_L_ units in almost all cases, whereas there were nevertheless ambiguous PSTHs which might or might not be classified as GBC-like if they had been encountered in physiological recording experiments (see Discussion).

#### Selection of model instances

The GBC model has six parameters (denoted as *M*_*E*_, *W*_*E*_, *A*_*E*_, *T*_*R*_, *T*_*A*_, and *S*_*A*_; see above for their definitions). In our preliminary simulations, we first fixed the number of excitatory inputs *M*_*E*_ = 20 and manually adjusted the other five parameters to obtain the desired GBC responses stated above. We call this instance the "baseline model". The parameters of the baseline model were: *M*_*E*_ = 20, *W*_*E*_ = 0.4 (ms), *A*_*E*_ = 0.4, *T*_*R*_ = 1.2 (ms) *T*_*A*_ = 0.3 (ms), *S*_*A*_ = 0.9; and it presented similar response patterns to the default GBC model (Table 1, bold). We next varied the model parameters around this baseline model using a coarse grid (4–6 values in the range between 0 and 200% of the baseline model parameters for each dimension) and examined which parameter combination led to a GBC-like response. A similar approach of combining a baseline model with systematic variation of parameters was used in previous studies [40,41].

In this coarse grid search, we obtained the count of accepted models for each parameter value (as shown in Fig 7B–7F). The resulting distribution of a parameter typically included a peak and decayed gradually around it. The steepness of peak and decay depended on each parameter and its adopted range. If the distribution was too narrow in comparison to the search range of the parameter grid, then we reduced the test range used for the next round of grid search. For example, instances with *A*_*E*_ ≤ 0.2 or *A*_*E*_ ≥ 0.6 almost never satisfied our criteria for GBCs (Fig 7C) and therefore the range for the input amplitude was reduced to exclude these values. If the width of the distribution seemed insufficient to include the tail part of the distribution, then we expanded the test range. By repeating this coarse grid search for several times, we gained knowledge on the relevant range for each parameter. After these attempts, we fixed the range of the model parameters used for this paper (Table 1). The range for the number of excitatory inputs *M*_*E*_, however, was determined from anatomical data of cat GBCs. Our range of 9–36 inputs covers 11 of the 12 measurements performed in [10]. In that study, the largest number of inputs reported was 69, which we considered exceptional and excluded from our simulations, as it was more than twice as many as the second largest count of 34. As shown in Fig 7B–7F, the distribution of the accepted PL_N_ model parameters showed a unimodal peak and covered the whole range we adopted, indicating that the selected parameter grid sampled the most important part of the parameter space. However, it is still probable that some parameter combinations outside of these ranges might result in GBC-like responses (see Discussion).

In total, we had 567,000 model instances that were further tested for their output measures (Table 1). We calculated the spontaneous and sound-driven responses of each of these instances and judged them using the criteria described in previous sections. We first measured the spontaneous rate (SR), high-frequency-driven rate (DR) and irregularity (CV') as well as low-frequency-driven phase-locking (VS) and entrainment (EI) to select candidate GBC instances (e.g., Fig 5B and 5C merged into 5D). We then tested their PSTHs in response to high frequency stimulation to obtain the final population of 7520 PL_N_ instances that satisfied all our criteria (Fig 5E). We repeated the same procedure but with a lower driven rate criterion (see above) to obtain an On_L_ population of 4094 instances.

### Parameter dependence

#### Connectivity

To study the distributions of the GBC-like model instances in our 6-dimensional parameter space grid, we performed a connectivity analysis [68]. Two model instances are *connected* if there is a path between them on the grid that satisfies the following: a *path* is a sequence of *neighboring* points (see next paragraph) in the grid space; and all the instances in the path are members of the same class (i.e., PL_N_-type models in our case). We used the Matlab function "bwconncomp" (MathWorks, Natick, MA) to calculate connectivity.

Two model instances that differ by one grid-step in one dimension are called *one-dimensional neighbors*. In the six-dimensional space, one instance has twelve one-dimensional neighbors (i.e., two directions along each of six dimensions). The *n-dimensional neighbor* of a model instance is defined as the set of instances that are reached by changing at most *n* parameters (out of six) to values that are next to the original parameter value. We used one-, two- and three-dimensional neighbors for our connectivity analysis; using higher-dimensional neighbors yielded the same results as for three-dimensional neighbors.

#### Convexity

As seen in Results, the distribution of the accepted model instances was likely to form one big cluster. Even though, the shape of the distribution may be skewed in the parameter space. To test this possibility, we performed a convexity analysis. We randomly sampled 3500 pairs from the population of our PL_N_ instances; for each pair, we made three *intermediate instances* by assigning parameters calculated with an interpolation between the parameter values of the original pair. Our criteria for a PL_N_ unit were then applied to each intermediate instance: namely, we calculated the spontaneous rate, high-frequency sound-driven responses (rate, CV', and PSTH) to 7000 Hz to at 70 dB, and low-frequency sound-driven responses (VS and EI) to 350 Hz at 70 dB. If the parameter distribution were totally convex, 100% of the intermediate instances would be categorized as PL_N_.

#### Input parameter correlations

In neuronal modeling, two model instances whose parameters are substantially different from each other can still yield similar outcomes, because some parameters may compensate with each other [42]. To reveal intercorrelations of the six parameters of the model, we calculated the correlation coefficients between each two parameters of our GBC population (Fig 8). A positive correlation coefficient indicates that an increase of one parameter can be (partly) counteracted by an increase of the other parameter to keep the output of the model largely unchanged. A negative correlation implies that the two parameters concerned may play similar roles in characterizing the response characteristics of the GBC units.

#### Input-output regressions

In order to investigate the relationship between the input parameters and output measures, we fitted linear, quadratic, and cubic functions of the model parameters to the outputs of our GBC instances (Fig 10). We used the six parameter variables (*M*_E_, *W*_E_, *A*_E_, *T*_R_, *T*_A_, *S*_A_) for the input and five spiking measures (SR, DR, CV', VS, EI) for the output. Before calculating the fits, we "z-scored" each input variable, by subtracting its mean and normalizing with its standard deviation, to handle all the parameters on a common scale [41]. Then the output variables except CV' were converted into log(SR), log(DR), log(1-VS), and log(1-EI), since we found that these conversion slightly improved polynomial fitting results.

The linear fit had six variables, while the quadratic fit had additional 21 variables (6 × each parameter squared + 15 pairs chosen out of the six). The cubic fit had 83 variables in total (6 linear, 21 quadratic, and 56 cubic). For the linear and quadratic fits, we performed an additional analysis to determine the relative contribution of each variable (or each pair of two variables). Starting from a constant term, we added one variable at each time and calculated how much more variance of the output was explained by this added variable. The order of addition generally affects the resulting gain of variance explained [41]. We therefore repeated the calculation 720 times for the linear fit (using all possible orders of the six parameters) and 40000 times for the quadratic fit (using orders randomly selected out of about 5 × 10^19^ possibilities), and calculated the mean gain obtained for each parameter. For the quadratic fit, we used the linear fit as the starting point, to ensure that no quadratic term was added without having its linear component in the fit.

#### Code and data availability

Matlab implementation of the model and numerical data for the simulated GBC population are available online at https://github.com/pinkbox-models.
